# Homeostatic structural plasticity – a key to neuronal network formation and repair

**DOI:** 10.1186/1471-2202-15-S1-P17

**Published:** 2014-07-21

**Authors:** Markus Butz, Arjen van Ooyen

**Affiliations:** 1 Simulation Lab Neuroscience, IAS, Jülich Aachen Research Alliance, Forschungszentrum Jülich; 2 Computational Neuroscience Group, Neuroscience Campus Amsterdam, VU Universiteit Amsterdam

## 

Homeostasis in electrical activity is a guiding principle for network formation and reorganization throughout life. Traditionally homeostatic plasticity is thought to adapt the weight of existing synapses so that postsynaptic firing rates are regulated towards or maintained at a desired set-point. However, neither during development nor in adulthood the connectivity structure of sparsely connected brain networks is fixed but continuously rewires. Here, we summarize our most recent modeling results showing that homeostasis in electrical activity can drive network formation during development as well as network reorganization after focal retinal lesions.

For both studies, we applied our model of structural plasticity (MSP) [1,2] in combination with a Gaussian-shaped kernel function for synapse formation, making the formation of connection between adjacent neurons more likely than between distant neurons. The novelty of MSP is that it represents a synapse not just as a weighted link between neurons but as consisting of two merged synaptic elements, an axonal and a dendritic element. Both develop individually dependent on the average electrical activity of the host neuron. Vacant synaptic elements will randomly merge in a distance-dependent manner (kernel function) to form a synapse or can break apart again, once a synaptic element is deleted again later on.

Interestingly, during network formation neurons started to form longer connections than expected from the kernel function as soon as they approached a homeostatic equilibrium in electrical activity making networks more efficiently connected as networks formed without a homeostatic rule for synapse formation [2]. Our theoretical findings were in striking accordance with experimental observations from developing dissociated cell cultures in which long-range connections also appeared around the time cell cultures reached a homeostatic equilibrium in neuronal activity.

We also applied MSP to restore neuronal activities after a focal loss of input [1]. Although, MSP is operating purely locally on the level of the individual neuron, a highly spatially and temporally ordered network reorganization emerged: First, neurons at the border of the area deprived of input formed a demand for additional horizontal inputs (from within the network) and were served by neurons sprouting axons from the intact surrounding. As soon neurons at the border had restored their level of activity by forming additional synapses, the frontier between neurons of normal and low activity levels moved more to the center of the input deprived area which sequentially “healed” like a wound from the outside to the inside. The course of network reorganization in our model matches the closing of a lesion projection zone in the visual cortex after focal retinal lesions in adult mice and monkeys. Remarkably, we can explain the experimental findings after focal retinal lesions including functional retinotopic mapping by structural plasticity alone. Neither STDP nor any other Hebbian form of plasticity was required for functional network reorganization in our model.

This work broadens our understanding of brain plasticity as observes forms of plasticity that go beyond mere Hebbian synaptic weight plasticity. Especially structural plasticity acts on long timescales from days to weeks and months that are crucial for brain development and network repair after brain lesions and so far hardly respected by computational neuroscience.
